# Trends and Focus of Machine Learning Applications for Health Research

**DOI:** 10.1001/jamanetworkopen.2019.14051

**Published:** 2019-10-25

**Authors:** Brett Beaulieu-Jones, Samuel G. Finlayson, Corey Chivers, Irene Chen, Matthew McDermott, Jaz Kandola, Adrian V. Dalca, Andrew Beam, Madalina Fiterau, Tristan Naumann

**Affiliations:** 1 Department of Biomedical Informatics, Harvard Medical School, Boston, Massachusetts; 2 Harvard Medical School, Boston, Massachusetts; 3 Predictive Health Care Group, University of Pennsylvania Health System, Philadelphia; 4 MIT Computer Science and Artificial Intelligence Lab, Boston, Massachusetts; 5 Department of Medicine, Imperial College London, London, United Kingdom; 6 Department of Epidemiology, Harvard T.H. Chan School of Public Health, Boston, Massachusetts; 7 College of Information and Computer Sciences, University of Massachusetts, Amherst; 8 Microsoft Research, Redmond, Washington

## Abstract

**Importance:**

The use of machine learning applications related to health is rapidly increasing and may have the potential to profoundly affect the field of health care.

**Objective:**

To analyze submissions to a popular machine learning for health venue to assess the current state of research, including areas of methodologic and clinical focus, limitations, and underexplored areas.

**Design, Setting, and Participants:**

In this data-driven qualitative analysis, 166 accepted manuscript submissions to the Third Annual Machine Learning for Health workshop at the 32nd Conference on Neural Information Processing Systems on December 8, 2018, were analyzed to understand research focus, progress, and trends. Experts reviewed each submission against a rubric to identify key data points, statistical modeling and analysis of submitting authors was performed, and research topics were quantitatively modeled. Finally, an iterative discussion of topics common in submissions and invited speakers at the workshop was held to identify key trends.

**Main Outcomes and Measures:**

Frequency and statistical measures of methods, topics, goals, and author attributes were derived from an expert review of submissions guided by a rubric.

**Results:**

Of the 166 accepted submissions, 58 (34.9%) had clinician involvement and 83 submissions (50.0%) that focused on clinical practice included clinical collaborators. A total of 97 data sets (58.4%) used in submissions were publicly available or required a standard registration process. Clinical practice was the most common application area (70 manuscripts [42.2%]), with brain and mental health (25 [15.1%]), oncology (21 [12.7%]), and cardiovascular (19 [11.4%]) being the most common specialties.

**Conclusions and Relevance:**

Trends in machine learning for health research indicate the importance of well-annotated, easily accessed data and the benefit from greater clinician involvement in the development of translational applications.

## Key Points

**Question:**

What topics are researchers in machine learning focused on and what methods and data sets do they use?

**Findings:**

This qualitative analysis of 166 accepted manuscript submissions to the Third Annual Machine Learning for Health workshop at the 32nd Conference on Neural Information Processing Systems found that easy-to-access, well-annotated data increased machine learning research within specific health domains (58.4% of submissions). Clinicians were involved in a small amount of machine learning for health (34.9% of submissions).

**Meaning:**

This analysis suggests that the interdisciplinary field of machine learning for health may be accelerated by easy-to-access, well-annotated data and would benefit from greater clinician involvement to develop into translational applications.

## Teaser

This qualitative study analyzes submissions to a popular machine learning for health workshop to assess the current state of health research, including areas of methodologic and clinical focus, limitations, and underexplored areas.

## Introduction

Machine learning for health care is a rapidly growing interdisciplinary field gaining interest in academia and practice.^1,2,3,4,5,6^ The purpose of this work was to perform quantitative and qualitative evaluations of the state of machine learning for health research. As the field evolves, analyses can elucidate research trends, behaviors, and future opportunities. In this work, we performed an analysis using the submitted and accepted manuscripts from the recent Third Annual Machine Learning for Health (ML4H) workshop at the 32nd Conference on Neural Information Processing Systems (NeurIPS) on December 8, 2018.^7^

The ML4H offers a unique opportunity to examine the trends of the field for 2 reasons. First, as a workshop, ML4H accepts manuscripts ranging from preliminary work indicative of ongoing research to fully complete studies, which subsequently are featured in full conferences or journals. This approach yields a more complete picture of the field than would be obtained by analyzing the output of a full conference alone. Second, ML4H is a large venue—in 2018, ML4H received 240 submissions from researchers applying machine learning to health, of which 80 were accepted for full poster presentations and an additional 86 were accepted for digital acceptances. By comparison, other prominent machine learning for health venues, including Medical Imaging Meets NeurIPS (n = 32),^8^ *Biomedical Natural Language Processing* Workshop of the Association of Computational Linguistics (n = 26),^9^ Machine Learning for Healthcare (n = 31),^10^ and the International Workshop on Data Mining in Bioinformatics (n = 42),^11,12,13^ had anywhere from 26 to 42 accepted manuscripts. Thus, ML4H’s large scale offers the opportunity to examine current trends, both quantitatively and qualitatively, within machine learning for health care.

Our analysis included 4 primary components. First, we conducted an expert review of all accepted submissions across a battery of criteria, including what data sets are used, how extensively technical authors directly collaborate with clinicians, and what clinical tasks and diseases are most studied. Second, beyond analyzing the content of the manuscripts themselves, we also analyzed how the pool of authors who submitted manuscripts to ML4H compare with the authors with manuscripts at the main NeurIPS conference. Third, we performed computational modeling of the content of the manuscripts. We found that the resultant topics appeared to be associated with meaningful research areas and tools. We made this topic breakdown accessible publicly through a web application, allowing readers and authors to see how various manuscripts relate to one another. Fourth, we concluded with a summary of the key themes and ideas present at the ML4H workshop and what these imply for the field on the whole.

## Methods

### Researcher Characteristics and Ethical Considerations

This study is a retrospective analysis of accepted and publicly disclosed research. There was no intervention, interaction with researchers, or use of private information to perform the analyses in this study. This study does not meet the definition of human research under the US Department of Health and Human Services protection of human subject regulations. Data were not deidentified and are publicly available. The study organizers all actively perform machine learning for health research, participated in organizing the workshop, and were involved in the acceptance process of submission for the workshop. All analyses should be considered in this context and the stated goal of the workshop: “moving beyond supervised learning.” The analyses in this study followed the Standards for Reporting Qualitative Research (SRQR) reporting guideline.^14^ Each portion of the analyses was made available to all authors, researchers did not perform rubric review on manuscripts for which they had conflicts of interest, and source code for topic analyses has been made publicly available.^15^

### Included Submissions

Organizers from the ML4H workshop at NeurIPS 2018 accepted a total of 166 manuscripts, including 16 selected for spotlight talks, 64 selected for poster presentations, and an additional 86 that were digitally accepted to be included on the workshop website. Rejected manuscripts were not included in the analysis; these manuscripts remain anonymous per the double-blind review process.

### Qualitative Rubric Review

Expert reviewers (B.B-J., C.C., I.C., M.M., J.K., M.F., and T.N.) evaluated each manuscript submission against a rubric, including 19 topics (eTable 1 in the Supplement). Structured answers were provided where possible, and free-text answers were analyzed to identify trends and synonyms across different manuscripts.

### Statistical Analysis

#### Author Analysis

We examined information on the authors of all accepted manuscripts. Author affiliations were self-identified during the submission process. As a comparison, we analyzed the authors for the NeurIPS 2018 conference main track. Affiliations of the NeurIPS authors were not provided and therefore not used; instead, only author names were analyzed. When comparing the authors across the ML4H workshop and the NeurIPS 2018 main track, we considered names to be matching if they had up to an edit distance of 3. Specifically, if 2 names were not an exact match but would become an exact match with at most 3 insertions, deletions, or substitutions, we considered them a match. This approach enabled us to identify an author even if small changes were made to their name, such as the presence or absence of diacritics.

#### Topic Modeling

In addition to the rubric-based thematic analysis, we built a topic model using latent Dirichlet allocation (LDA)^16,17^ from the raw text of the corpus of submitted manuscripts. This model treats individual submissions as having been generated from a distribution over topics, with topics in turn being modeled as distributions over words. We took a standard approach to tokenization, removing punctuation, digits, and special characters. We also removed stop words from the SMART English-language stop word list^18^ and words that appeared fewer than 5 times in the corpus. We built a topic model, selecting *k* = 12 topics by minimizing perplexity on a 90:10 split at the document level (eFigure in the Supplement). We present a visualization of the resulting 12 topic models trained on the entire corpus of accepted manuscripts using LDA via the LDAvis R package.^19^

#### Access to Data and Source Code

All the data used in our analyses were extracted from publicly visible web pages. Code for recreating the data and replicating all models and analyses is publicly available on Github.^15^

## Results

### Clinical Collaboration

Of the 166 submissions evaluated, 58 (34.9%) involved a collaboration with clinicians in the form of collaborating consultants (6 [3.6%]), authors (39 [23.4%]), or primary authors (13 [7.8%]). For clinical practice submissions, 35 (21.1%) involved a collaboration with clinicians in the form of collaborating consultants (4 [2.4%]), authors (25 [15.1%]), or primary authors (6 [3.6%]). The reviewers categorized each manuscript based on task subject (eg, biomedical research, clinical operations, clinical practice, policy, core methods development, and other). Among manuscripts studying clinical practice, 83 projects (50.0%) included clinical collaborators.

### Data Used for Research

A total of 97 data sets (58.4%) used in submissions were public (Table 1). This was also true about each category, with the exception of speech data sets for which only 1 of 6 data sets (16.9%) was public. Only 20 submissions (12.0%) used more than 1 data source, and only 9 (5.4%) trained on 1 data set and evaluated a different external data set.

**Table 1.** Data Sets and Data Access for Each Type of Data

Data Type	Total No. of Data Sets	No. (%) of Data Sets	
		Public or Apply for Access	Private, Institutional Access, Self-collected, or Synthetic (Not Shared)
Total	166	97 (58.4)	69 (41.6)
Structured	54	28 (51.9)	26 (48.1)
Image	39	28 (71.8)	11 (28.2)
Text	34	19 (55.9)	15 (44.1)
Biological sequence	10	8 (80.0)	2 (20.0)
ECG	10	6 (60.0)	4 (40.0)
EEG	7	4 (57.1)	3 (42.9)
Speech	6	1 (16.7)	5 (83.3)
Video	6	3 (50.0)	3 (50.0)

Abbreviations: ECG, electrocardiography; EEG, electroencephalography.

A total of 127 data sets (76.5%) used were structured, image, or text data sets (Table 1). Following these categories of data sets were biological sequences, electrocardiographic (ECG) and electroencephalographic (EEG) waveforms, speech, and video data sets. A notable confounder in this ordering may be that the NeurIPS computational biology workshop did not occur in 2018, meaning that we may have received more biological sequence submissions than otherwise expected.

Several data sets were used in multiple submissions. For example, the Physionet^20^ data sets (MIMIC-III^21^ and eICU^22^), which were used in 18 submissions, represented 34.6% of the structured data set use. Another commonly used data set was the Alzheimer’s Disease Neuroimaging Initiative, which was used in 10 submissions primarily for images and biological sequences (eg, genetic variants). These 2 data sources have publicly available application processes. The UK Biobank, although relatively new and with a longer approval process, was used in 3 submissions. Data sets used that are publicly available or have publicly available registration processes are listed in eTable 2 in the Supplement.

### Studied Clinical Conditions and Tasks

The accepted manuscripts studied an array of clinical conditions, as detailed in Table 2. Clinical practice was the most common application area (70 manuscripts [42.2%]), with the most prevalent category (25 [15.1%]) being conditions related to the brain and mental health (neurologic, psychological, and cognitive disorders). A total of 21 manuscripts (12.7%) were related to cancer, most often its diagnosis. Cardiovascular conditions were also well represented (19 [11.4%]), as was diabetes (10 [6.0%]). Seven methods (4.2%) were specifically designed to address multiple conditions. A total of 43 manuscripts (25.9%) did not address any condition in particular and instead introduced general method improvements. Several clinical conditions were addressed by 1 manuscript each: colectomy surgery, genetic diseases, childhood obesity, cystic fibrosis, drug reactions, liver disease, sleep disorders, glaucoma, facial expressions and injury prevention, rare diseases, epidemic processes, and aging.

**Table 2.** Clinical Conditions

Condition	No. (%) of Manuscripts^a^
Brain and mental health	25 (15.1)
Oncology	21 (12.7)
Cardiovascular	19 (11.4)
Diabetes	10 (6.0)
Pregnancy and natality	6 (3.6)
Pulmonary	6 (3.6)
ICU	5 (3.0)
Infection	5 (3.0)
Mobility and skeletal conditions	4 (2.5)
Mortality	3 (1.8)
Vocal disorder	3 (1.8)
Quality of care or quality of life	2 (1.2)
Hemorrhage	2 (1.2)
Addiction, smoking, opioid	2 (1.2)
Multiple	7 (4.2)
Other	12 (7.8)
NA	43 (25.9)

Abbreviations: ICU, intensive care unit; NA, not applicable.

^a^ Some manuscripts addressed more than 1 condition (percentages will not equal 100).

We also analyzed the clinical tasks addressed in the manuscripts. Diagnostication was by far the most prevalent clinical task studied (57 [34.3%]), with prognostication, subtyping, and treatment planning also receiving attention (eTable 3 in the Supplement). A total of 61 manuscripts (36.8%) did not focus on a specific clinical task.

### Machine Learning Tasks and Methods

This year’s theme of the ML4H workshop was moving beyond supervised learning in health care. Nevertheless, more than half of the manuscripts addressed classification problems. Only 11 (6.6%) addressed clustering, and 8 (4.8%) addressed regression (eTable 4 in the Supplement). Representation learning was the end goal in 8 manuscripts (4.8%) and generative models in 6 (3.6%), although both methods appeared frequently as a means to performing classification. Only 4 manuscripts (2.4%) studied forecasting, causal analysis, and survival analysis. Nine manuscripts (5.4%) undertook multiple tasks.

Methodologically, 115 of 166 manuscripts (69.3%) used deep learning models (eTable 5 in the Supplement). Convolutional neural networks (39 [23.5%]) and other neural networks (30 [18.1%]) were the most commonly used, whereas models in the recurrent neural network family, such as long short-term memory (LSTM) networks and gated recurrent units, accounted for 25 manuscripts (15.1%). Generative adversarial networks were used by 8 manuscripts (4.8%), as were tree-based models. Graphical models were only used in 7 cases (4.2%), whereas linear models and support vector machines accounted for 9 manuscripts (5.4%). Gaussian processes were used in 6 manuscripts (3.6%) and latent variable models in 4 manuscripts (2.4%).

Of the surveyed manuscripts, 74 (44.6%) tackled new methods. Surprisingly, only 40 manuscripts (24.1%) documented the application of existing state-of-the-art techniques on the tasks that were undertaken. The reduced use of recent methods is associated with authors relying on older, established methods when no novel methodologic contributions were made and when new algorithms were presented. In the latter case, for some manuscripts, only the new methods were tested.

### Author Analysis

 The ML4H workshop had 604 unique authors who had submitted manuscripts, with a mean (SD) of 1.13 (0.45) manuscripts per author. Each manuscript had a mean (SD) of 4.10 (2.07) authors and 2.14 (1.70) affiliations. The per-manuscript numbers were higher than the main NeurIPS conference, where there were 3.79 (1.65) authors per manuscript among 3127 unique authors. There were 32 overlapping authors between the main conference track and the ML4H workshop.

### Topic Modeling

**Figure 1.**
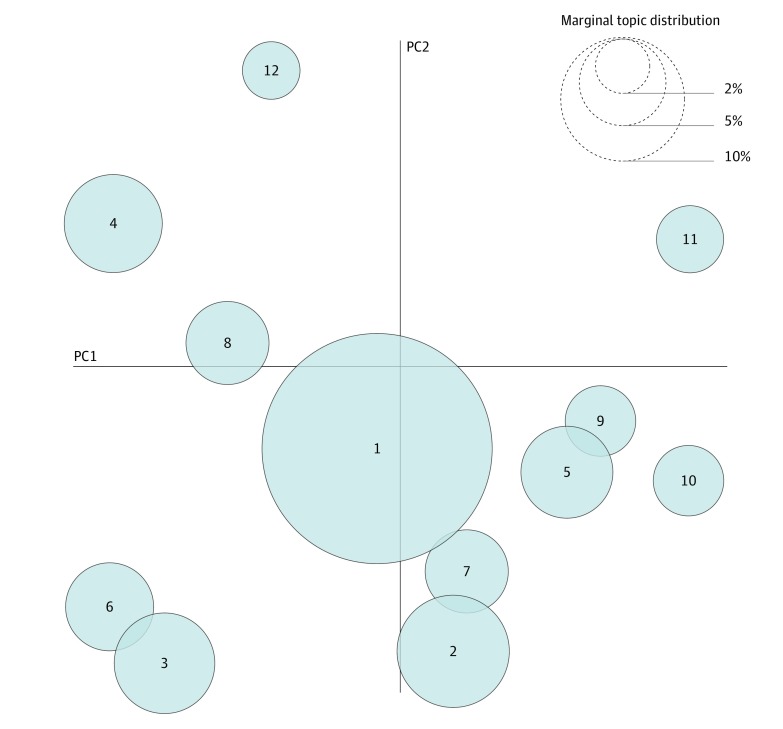
Visualization of 12-Topic Model Trained on Third Annual Machine Learning for Health (ML4H) Workshop Manuscripts Principal component (PC) projection of 12 topics learned from ML4H manuscripts. An interactive version can be viewed online.^23^

**Figure 2.**
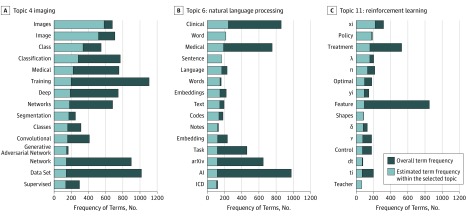
Selected Topics Representing Application Domains and Methods From the Topic Model Trained on Third Annual Machine Learning for Health (ML4H) Workshop Manuscripts An interactive version can be viewed online.^23^ AI indicates artificial intelligence; *ICD*, *International Classification of Diseases*. dt, ti, xi, and yi are equation variables common in reinforcement learning algorithms.

Most of the 12 topics resulting from the LDA model were broadly interpretable along methodologic or substantive clinical domain axes (Figure 1). The first most dominant topic (as measured by marginal probability) was a generic machine learning/artificial intelligence topic (keywords: *data, model,* and *learning*), and the second was a generic medical topic (keywords: *patients, health, disease, risk,* and *treatment*), but the remaining topics appeared to represent distinct methods or application areas. The third topic involved temporality and techniques for longitudinal modeling and biosignals (keywords: *time, LSTM, patients, series, recurrent, signal, ECG, speech,* and *sequence*), and the fourth topic appeared focused on imaging (keywords: *images, classification, deep, segmentation, convolutional, generative adversarial network,* and *supervised*) (Figure 2A). The fifth topic seemed to primarily represent generative modeling (keywords: *latent, distribution, space, generative, variational, reconstruction, prior, posterior,* and *variational autoencoder*), and the sixth topic represented natural language processing (keywords: *word, medical, sentence, language, embeddings, corpus,* and *LSTM*) (Figure 2B). The seventh topic appeared marked by terms from survival analysis and patient stratification (keywords: *survival, cluster, analysis, time, risk, event, progression, observations, clustering,* and *cancer*), whereas the eighth topic seemed focused on neuroimaging (keywords: *brain, EEG, convolutional, image, Alzheimer, magnetic resonance imaging, Alzheimer disease, neural, sleep, dementia, age, volumes, scans, dice,* and *cognitive*). The ninth topic (keywords: *RNA*) seemed focused on genomics and the 10th topic (keywords: *genetic, causal, effect, trait, phenotypes, biobank,* and *single-nucleotide polymorphisms*) on genetics. The 11th topic appeared dominated by controls and sequential decision-making (keywords: *policy, treatment, λ, π, optimal, control,* and *teacher*) (Figure 2C) and the 12th by privacy and security (keywords: *privacy, noise, algorithm, loss, quality, training, distributed, secure, clean,* and *distance*).

Visualization of all 12 topics via multidimensional scaling revealed global and local structure. Globally, the first principle component appeared to correspond at low values to topics that heavily involve deep learning (computer vision, natural language processing, time series, neuroimaging, and privacy and security) and at high values to topics that involve statistics or theoretical derivations (generative modeling, genetics, genomics, and controls/reinforcement learning). Local structure highlighted topics that tend to share related methods, such as natural language processing and time series (clusters 3 and 6) and generative modeling, genomics, and genetics (clusters 5, 9, and 10).

## Discussion

The workshop highlighted a broad range of trends, challenges, and key questions in the field of machine learning for health care by means of the accepted manuscripts as well as the invited speakers and panel discussion. Manuscripts accepted at the ML4H workshop included a range of authors, medical domains, and clinical tasks. Given that only 32 ML4H authors (5.3%) participated in the main track at NeurIPS and 58 submission (34.9%) actively involved clinicians, the workshop appeared to attract a broader distinct community from that of NeurIPS. Future work will attempt to more directly assess the project stages at which clinicians are involved (eg, study design, methods, interpretation, and discussion).

An objective yet informative component of machine learning manuscripts are the data sets they use. Several trends emerge from our analysis of the data sets leveraged at ML4H. First, publicly available medical data sets appear to be valuable resources for machine learning researchers. A total of 97 manuscripts (58.4%) accepted at the workshop used public data. Although this is far lower than what has been observed for other subfields of machine learning, it is slightly higher than a prior analysis^24^ found for machine learning for health care more broadly. Although this observation is encouraging—public data enhance reproducibility—we also found that the 3 most common data sets used (MIMIC, eICU, and Alzheimer’s Disease Neuroimaging Initiative) alone accounted for 28 manuscripts (16.9%). Although this observation underscores the tremendous value and interest in open clinical data sets, this disproportionate use of few selected data sets may cause results from the field to become biased toward a small number of currently available data sources.

A related observation is the dearth of validation within manuscripts, with only 9 manuscripts (5.4%) validating a model on a separate data set from that on which they were trained. This troubling finding is consistent with retrospective analyses^6,25^ of manuscripts from the literature. Taken together, the continued coupling of exclusively internal validation with poor demographic, spatial, and temporal diversity is likely to lead to poor generalization to new institutions as well as susceptibility to data set shift. In this light, these findings imply a strong need for continued efforts to collect and annotate diverse public health care data sets, a finding echoed by other analyses of this space.^24^

Data modality studies also indicate significant specialization of the field into structured data, imaging, and text analysis, which made up 127 ML4H submissions (76.5%). Although this finding is not exactly surprising, it indicates a dearth of analyses of other data types. Waveform data, in particular, present a compelling clinical use case (eg, to characterize EEGs^26^ and to detect rare heart arrhythmias^27^) but are understudied by our analysis. Video data, a major area of study in generic machine learning,^28^ were used little here, although this may be because of the additional privacy implications of working with this modality.

Despite the fact that our workshop theme was moving beyond supervised learning in health care, supervised learning manuscripts were not formally discouraged in the call for manuscripts; we noted that 100 manuscripts (60.2%) focused exclusively on supervised tasks, such as classification, regression, or segmentation (eTable 3 in the Supplement). Without directly surveying the authors for explanations, it is difficult to provide a definitive explanation. However, we speculate from experience that this may be in part because supervised learning projects are much simpler to design and defend, particularly in health care: supervised learning by definition lends itself to clear-cut tasks with objective evaluations (eg, accuracy in a diagnosis or prediction) and are simple to understand and explain. By the same token, many data sets come with annotations that can be used for supervised prediction (which may be appealing to nonclinicians), and machine learning frameworks, such as TensorFlow,^29^ PyTorch,^30^ and scikit-learn,^31^ generally require little modification to implement and evaluate supervised learning (which may be appealing to new and established clinicians seeking simple implementation). On a related note, we observe that simpler-to-use data types, such as structured data (54 manuscripts [32.5%]), images (30 [23.5%]), and text (34 [20.5%]), are used far more frequently than data types that require more technical expertise to wrangle and clinical expertise to interpret, such as biosignals, including ECG (10 manuscripts [6.0%]) and EEG (7 [4.2%]), speech (6 [3.6%]), and video (6 [3.6%]). We view this as a great opportunity for interdisciplinary collaborations, which may be necessary to lower the barrier to data types (biosignals) and data tasks (representation learning and generative modeling) that appear more daunting to those without clinical expertise and/or technical experience working with similar data.

The topic modeling analysis identified meaningful clusters of manuscripts in terms of methods and areas of applications. The unsupervised clustering qualitatively highlights application areas that are becoming increasingly intertwined because of shared methods. For example, one trend is that deep recurrent networks have contributed to a methodologic synergy between longitudinal electronic health record data or natural language processing and biosignals. This trend is apparent from the collocation of topics 3 (*time, LSTM, patients, series, recurrent, signal, ECG, speech,* and *sequence*) and 6 (*word, medical, sentence, language, embeddings, corpus,* and *LSTM*). At the same time, it is notable that the largest global structure in our topic modeling was the separation of fields increasingly dominated by deep learning manuscripts (eg, computer vision, natural language processing, and biosignals) from more statistical or theoretically focused fields (eg, latent variable modeling, genetics, genomics, and sequential decision making). By highlighting such substructure within medical machine learning, we hope to inspire new collaborations among researchers: both between researchers in fields such as natural language processing and structured hospital data, which appear to be naturally converging, and also between subfields such as bayesian and deep learning, which appear to be currently well separated and may benefit from active efforts to cross-fertilize.

In addition, authors across all accepted submissions appeared to systematically favor the term *machine learning* to *artificial intelligence* (*machine learning* and acronyms appeared 4841 times and *artificial intelligence* and acronyms appeared 1322 times). This finding is consistent with our experience that technical researchers in this community tend to describe their work in terms of the methods used rather than the umbrella term of *artificial intelligence*. Given that work published in the medical literature routinely refers to the approach as *artificial intelligence*,^32,33^ this could contribute to a language barrier between clinicians and traditional machine learning researchers.

### Limitations

This study was a retrospective analysis of manuscripts accepted to the ML4H workshop at NeurIPS, which presents several limitations to the analysis, notably the potential for ascertainment and selection biases. Inclusion in this study requires both submission and acceptance of a manuscript to the workshop. In particular, the general NeurIPS conference caters to a technical computer science and machine learning audience, which may result in an enrichment of manuscripts that present technical novelty. In future years, it will be possible to compare trends over time for both clinical and methodologic areas.

## Conclusions

Machine learning for health is an intensely interdisciplinary field. Effectively deployed clinical systems require an alignment of data availability, clinical expertise, technical soundness, and economic incentives. Invited discussion at the ML4H workshop highlighted a number of common hindrances to achieving the stated theme of implementing advanced machine learning methods in health care. The requirement for collaboration among key players before project development is the key means of achieving these goals was a recurring theme.

Analysis of accepted manuscripts highlighted the breadth of clinical tasks for which machine learning can be significant. Unsupervised topic modeling suggests a nascent opportunity for new collaborations within the field because deep learning methods have enabled technical convergence among different fields. As highlighted by expert panelists, the same analysis highlights that there may be additional room for technical collaboration among researchers working on subfields that currently are generally disconnected and may benefit from more explicit efforts for collaboration. Finally, manuscripts in general appeared limited in their validation on external data, which may be attributable to an absence of diverse public data sets or a lack of demand for external validation from researchers and reviewers. Regardless of the cause, the need for more active investment into robust machine learning is required as a safeguard against poor prospective performance in new settings.

Although many challenges were discussed at the workshop and in the submitted manuscripts, there was consensus about the enormous potential to solve important health care problems using machine learning. Conversely, health care continues to evolve as one of the most important application areas for machine learning. For the machine learning for health community to realize its full potential, serious and sustained collaboration among researchers from both communities will be required.

## Notes

Supplement. **eTable 1.** Description of Rubric Categories Evaluated **eTable 2.** Data Sets That Are Either Publicly Available or Available After Registration **eTable 3.** Clinical Tasks **eTable 4.** Primary Machine Learning Tasks **eTable 5.** Primary Machine Learning Method Used **eFigure.** Number of Topics (k) vs Perplexity as Measured on a Hold-out Set of 10% (n=17) of the Papers Showing Perplexity Minimized at k=12 **eReferences**

## References

[1] Beam AL, Kohane IS Big data and machine learning in health care. JAMA. 2018;319(13):-. doi:10.1001/jama.2017.18391 29532063

[2] Ching T, Himmelstein DS, Beaulieu-Jones BK, Opportunities and obstacles for deep learning in biology and medicine. J R Soc Interface. 2018;15(141):20170387. doi:10.1098/rsif.2017.0387 29618526 PMC5938574

[3] Naylor CD On the prospects for a (deep) learning health care system. JAMA. 2018;320(11):1099-1100. doi:10.1001/jama.2018.11103 30178068

[4] Jha S, Topol EJ Adapting to artificial intelligence: radiologists and pathologists as information specialists. JAMA. 2016;316(22):2353-2354. doi:10.1001/jama.2016.17438 27898975

[5] Hinton G Deep learning: a technology with the potential to transform health care. JAMA. 2018;320(11):1101-1102. doi:10.1001/jama.2018.11100 30178065

[6] Topol EJ High-performance medicine: the convergence of human and artificial intelligence. Nat Med. 2019;25(1):44-56. doi:10.1038/s41591-018-0300-7 30617339

[7] Antropova N, Beam AL, Beaulieu-Jones BK, Machine Learning for Health (ML4H) workshop at NeurIPS 2018. Preprint. Posted online November 17, 2018. arXiv 1811.07216.

[8] Medical Imaging Meets NeurIPS. Medical Imaging Meets NeurIPS. https://sites.google.com/view/med-nips-2018. Accessed September 2, 2019.

[9] Medical question answering—textual inference and question entailment in the medical domain. ACL-BioNLP’19 Shared Task. https://sites.google.com/view/mediqa2019. Accessed September 2, 2019.

[10] Machine learning for healthcare. Machine Learning for Healthcare website. https://www.mlforhc.org/. Accessed September 2, 2019.

[11] Machine learning for medicine and healthcare. https://mlmhworkshop.github.io/mlmh-2018/. Accessed September 2, 2019.

[12] BIOKDD’19. http://home.biokdd.org/biokdd19/. Accessed September 2, 2019.

[13] epiDAMIK: Epidemiology meets Data Mining and Knowledge Discovery. http://people.cs.vt.edu/~badityap/epidamik/. Accessed September 2, 2019.

[14] O’Brien BC, Harris IB, Beckman TJ, Reed DA, Cook DA Standards for reporting qualitative research: a synthesis of recommendations. Acad Med. 2014;89(9):1245-1251. doi:10.1097/ACM.0000000000000388 24979285

[15] Chivers C Topic analysis Github repository ML4H 2018. Github website. https://github.com/cjbayesian/ml4h_paper_2019. Accessed August 26, 2019.

[16] Xing EP, Jordan MI, Russell SJ, Ng AY Distance metric learning with application to clustering with side-information In: Becker S, Thrun S, Obermayer K, eds. Advances in Neural Information Processing Systems. Vol 15 Boston, MA: MIT Press*;* 2003:521-528.

[17] Blei DM, Ng AY, Jordan MI Latent Dirichlet allocation. J Mach Learn Res. 2003;3:993-1022.

[18] Lewis DD, Yang Y, Rose TG, Li F RCV1: a new benchmark collection for text categorization research. J Mach Learn Res. 2004;5(April):361-397.

[19] Sievert C, Shirley K LDAvis: a method for visualizing and interpreting topics In: Proceedings of the Workshop on Interactive Language Learning, Visualization, and Interfaces. Baltimore, MD: Association of Computational Lingustics; 2014:63-70.

[20] Goldberger AL, Amaral LAN, Glass L, PhysioBank, PhysioToolkit, and PhysioNet: components of a new research resource for complex physiologic signals. Circulation. 2000;101(23):E215-E220. doi:10.1161/01.CIR.101.23.e215 10851218

[21] Johnson AEW, Pollard TJ, Shen L, Lehman LH MIMIC-III, a freely accessible critical care database. Sci Data. 2016;3:160035. doi:10.1038/sdata.2016.35 27219127 PMC4878278

[22] Pollard TJ, Johnson AEW, Raffa JD, Celi LA, Mark RG, Badawi O The eICU Collaborative Research Database, a freely available multi-center database for critical care research. Sci Data. 2018;5:180178. doi:10.1038/sdata.2018.178 30204154 PMC6132188

[23] Machine Learning for Health—2018 interactive topic modeling. ML4H—2018 Topic Modelling. https://ml4health.github.io/2018/viz/. Accessed September 2, 2019.

[24] Mc Dermott MBA, Wang S Reproducibility in machine learning for health. https://openreview.net/pdf?id=HylgS2IpLN. Accessed September 20, 2019.

[25] Kim DW, Jang HY, Kim KW, Shin Y, Park SH Design characteristics of studies reporting the performance of artificial intelligence algorithms for diagnostic analysis of medical images: results from recently published papers. Korean J Radiol. 2019;20(3):405-410. doi:10.3348/kjr.2019.0025 30799571 PMC6389801

[26] Mirowski P, Madhavan D, LeCun Y, Kuzniecky R Classification of patterns of EEG synchronization for seizure prediction. Clin Neurophysiol. 2009;120(11):1927-1940. doi:10.1016/j.clinph.2009.09.002 19837629

[27] Spechbach H, Morel P, Ing Lorenzini K, Reversible ventricular arrythmia induced by dasatinib. Clin Case Rep. 2013;1(1):20-25. doi:10.1002/ccr3.5 25356203 PMC4184536

[28] Guo Y, Liu Y, Oerlemans A, Lao S, Wu S, Lew MS Deep learning for visual understanding: a review. Neurocomputing. 2016;187:27-48. doi:10.1016/j.neucom.2015.09.116

[29] Abadi M, Barham P, Chen J, Tensorflow: a system for large-scale machine learning In: Proceedings of the 12th Usenix Symposium on Operating Systems Design and Implementation*.* Berkeley, CA: USENIX; 2016:265-283.

[30] Paszke A, Gross S, Chintala S, Automatic differentiation in PyTorch. https://openreview.net/pdf?id=BJJsrmfCZ. Published October 2017. Accessed August 30, 2019.

[31] Pedregosa F, Varoquaux G, Gramfort A Scikit-learn: machine learning in Python. 2011 http://www.jmlr.org/papers/v12/pedregosa11a.html. Accessed September 20, 2019.

[32] Liang H, Tsui BY, Ni H, Evaluation and accurate diagnoses of pediatric diseases using artificial intelligence. Nat Med. 2019;25(3):433-438. doi:10.1038/s41591-018-0335-9 30742121

[33] Fernández-Ruiz I Artificial intelligence to improve the diagnosis of cardiovascular diseases. Nat Rev Cardiol. 2019;16(3):133. doi:10.1038/s41569-019-0158-5 30683888

