# Supplementary material for: Trends and Focus of Machine Learning Applications for Health Research
Source: JAMA Netw Open. 2019 Oct 25;2(10):e1914051. doi: 10.1001/jamanetworkopen.2019.14051 (PMC6822089; doi:10.1001/jamanetworkopen.2019.14051)
Supplement: Supplement. — eTable 1. Description of Rubric Categories Evaluated eTable 2. Data Sets That Are Either Publicly Available or Available After Registration eTable 3. Clinical Tasks eTable 4. Primary Machine Learning Tasks eTable 5. Primary Machine Learning Method Used eFigure. Number of Topics (k) vs Perplexity as Measured on a Hold-out Set of 10% (n=17) of the Papers Showing Perplexity Minimized at k=12 eReferences [file jamanetwopen-2-e1914051-s001.pdf]

Supplementary Online Content

Beaulieu-Jones B, Finlayson SG, Chivers C, et al. Trends and focus of machine learning applications for health research. *JAMA Netw Open*. 2019;2(10):e1914051. doi:10.1001/jamanetworkopen.2019.14051

eTable 1. Description of Rubric Categories Evaluated

eTable 2. Data Sets That Are Either Publicly Available or Available After Registration

eTable 3. Clinical Tasks

eTable 4. Primary Machine Learning Tasks

eTable 5. Primary Machine Learning Method Used

eFigure. Number of Topics (k) vs Perplexity as Measured on a Hold-out Set of 10% (n=17) of the Papers Showing Perplexity Minimized at k=12

eReferences

This supplementary material has been provided by the authors to give readers additional information about their work.

© 2019 Beaulieu-Jones B et al. *JAMA Network Open*.

eTable 1. Description of Rubric Categories Evaluated

**Rubric Category**	**Potential Values**	**Description**
Key Words	Free Text	Determined by author submission and reviewer determination.
Clinician Involvement	Main Authors, Authors, Consultants, None	Check if primary authors are clinicians. If not, check any other authors. If none, search for mentions of physician, clinician, doctor in text to see if collaborators consulted. If not, choose none.
Research Area	Biomedical Research, Clinical Operations, Clinical Practice, Policy, Core Methods Development, Other	Which area is the research aimed at improving?
Conditions Studied	Free Text	Which medical conditions were studied? (if any)
Clinical Tasks	Diagnostician, Prognostication, Subtyping, Biomarker Discovery, Treatment Planning, Other (with text description), NA	If a clinical task was performed, what was its purpose?
Machine Learning Task	Classification, Regression, Clustering, Representation Learning (Encoding / Embedding), Autoregressive Forecasting, Imputation, Generative Model (GAN, VAE or other), Causal Analysis, Other	What was the machine learning task performed?

© 2019 Beaulieu-Jones B et al. *JAMA Network Open*.

Machine Learning Methodology	Convolutional Neural Network, Long-short term memory network, Gated recurrent unit, Multilayer Perceptron, Other Recurrent Neural Network, Other Neural Network, Generative Adversarial Network, Linear Model, Tree-based Model, Nearest Neighbor Model, Gaussian Processes, Hidden Markov Model	What types of models / architectures were used to perform the task?
Develops New Machine Learning Methodology	Yes, No	Does the work develop a new machine learning methodology or apply an existing method?
Are the methods designed to address a particular common issue in using Machine Learning for Health?	No, Class Imbalance, Limited Training Data, Domain Adaptation, Weak Labels, Missing Data, Confounding, Other (with text description)	Are the methods designed to achieve better results using machine learning under conditions common to healthcare?
Were state of the art comparisons used (vs. strawman?)	Yes, No	Did the work compare results to state of the art methods and/or human performance? Or were only strawman comparisons made (if any)?
Datasets	Free Text	What datasets were used in the work?
Dataset Access	None, Public, Public Apply for Access, Institutionally Gated Apply for Access, Private, Self-Collected	Can others access the data used in the work?
**Datatype**	**Structured, Speech, Image, Video, Text, Genetic (and other structured biomarker), ECG, EEG**	**What type of data was used in the project?**

© 2019 Beaulieu-Jones B et al. *JAMA Network Open*.

Structured Covariates	Free Text	Were additional covariates provided for the dataset?
Sample Size	Free Text	How many samples were included in the dataset?
Feature Count	Free Text	How many features were present per sample?
Examined Multiple Datasets	Yes, No	Did the work analyze multiple datasets? (either through integration or evaluation)
Separate Evaluation Dataset	None, Test Set from Same Dataset, Test Set from External Dataset	Did the work evaluate results using a separate external dataset?
Interpretability Analyses Performed	Yes, No	Were any interpretability analyses performed?

© 2019 Beaulieu-Jones B et al. *JAMA Network Open*.

eTable 2. Data Sets That Are Either Publicly Available or Available After Registration

**Category**	**Datasets**
Image	Alzheimer's Disease Neuroimaging Initiative [1],<br>Dermnet Skin Disease Atlas [2],<br>Broad Bioimage Benchmark Collection [3],<br>Chest X-Ray8 [4],<br>ISBI-ISIC 2017 melanoma classification challenge [5],<br>ImageCLEF Medical Image Retrieval Task Test Collection [6],<br>Montgomery County X-ray Set [7],<br>Japanese Society of Radiological Technology [8]<br>Cancer Genome Atlas (MRI Collections) [9],<br>LITS: Leveraging Liver Tumor Segmentation Challenge [10],<br>Open Access Series of Imaging Studies (OASIS) Brains [11],<br>MIMC-CXR [12],<br>Indiana University Chest X-Ray [13],<br>Indian diabetic retinopathy image dataset (IDRiD) [14]
Structured (EMR, Registry, Claims etc)	MIMIC-III [15],<br>eICU [16],<br>Alzheimer's Disease Neuroimaging Initiative [1],<br>CDC Linked birth and infant death data [17] ,<br>MAGGIC [18],<br>Prostate, Lung, Colorectal and Ovarian Cancer (PLCO) [19],<br>Surveillance Epidemiology, and End Results (SEER) [20],<br>International Stroke Trial [21],<br>The Cancer Genome Atlas [22]<br>PRO-ACT Database [23]<br>MIMIC-CXR [12]
Text	i2b2 2010 challenge [24],<br>UK Biobank [25],<br>MIMIC-III [15],<br>eICU [16],<br>Framingham heart study [26],<br>Medline [27],<br>AskAPatient [28],<br>CADEC corpus [29],<br>FB15k-237 knowledge base completion [30],<br>NHANES [31],<br>The Cancer Genome Atlas [22],<br>CIHI [32]
Genetic	UK Biobank [25],<br>DeepArg [33],<br>Alzheimer's Disease Neuroimaging Initiative [1],<br>METABRIC [34],<br>The Cancer Genome Atlas [22],

© 2019 Beaulieu-Jones B et al. *JAMA Network Open*.

	PsychENCODE [35]<br>Pan-Cancer Analysis of Whole Genomes [36]
ECG	MIT-BIH [37],<br>Capture-24 [38]
EEG	The Human Connectome Project [39–41],<br>Temple University Hospital Seizure Detection Corpus [42],<br>UCI-EEG [43],<br>MIT-EEG [44],<br>Sleep-EDF [45]
Video	Parkinson@Home [46]
Protein	BindingDB [47]
Speech	Google Speech Commands [48]

© 2019 Beaulieu-Jones B et al. *JAMA Network Open*.

eTable 3. Clinical Tasks

**Clinical task**	**Paper Count (Percentage of total)**
Diagnostication	57 (34.3%)
Prognostication	16 (9.6%)
Subtyping	13 (7.8%)
Treatment Planning	10 (6.0%)
Biomarker Discovery	3 (1.8%)
Other	6 (3.6%)
NA	61 (36.8%)

Each paper can only be in one category. Percentage of the total number of papers shown in parentheses.

© 2019 Beaulieu-Jones B et al. *JAMA Network Open*.

eTable 4. Primary Machine Learning Tasks

**ML Task**	**Paper Count (Percentage of Total)**
Classification	86 (51.81%)
Clustering	11 (6.63%)
Regression	8 (4.82%)
Representation Learning	8 (4.82%)
Generative Model	6 (3.61%)
Forecasting	4 (2.41%)
Causal Analysis	4 (2.41%)
Survival Analysis	3 (1.81%)
Policy Learning	2 (1.2%)
Segmentation	2 (1.2%)
Imputation	2 (1.2%)
Multiple	9 (5.42%)
Other (feature selection, multiple instance learning, synthesis of minority class, image reconstruction, mixed effect modeling, summarization, dialogue tasks, concept normalization, automated measurement, NLP mapping, association tests.)	21 (12.65%)

Each paper can only be in one category. Percentage of the total number of papers shown in parentheses.

© 2019 Beaulieu-Jones B et al. *JAMA Network Open*.

eTable 5. Primary Machine Learning Method Used

**ML Methodology**	**Paper Count (Percentage of Total)**
CNN	39 (23.49%)
MLP	2 (1.2%)
Other NN	30 (18.07%)
LSTM	16 (9.64%)
GRU	4 (2.41%)
Other RNN	5 (3.01%)
GAN	8 (4.82%)
HMM	5 (3.01%)
Other Graphical Model	2 (1.2%)
Tree-based Model	8 (4.82%)
Linear Model	7 (4.22%)
SVM	2 (1.2%)
Gaussian Processes	6 (3.61%)
LVM	4 (2.41%)
Nearest Neighbor Model	5 (3.01%)
Reinforcement Learning	2 (1.2%)
Transfer Learning	2 (1.2%)

© 2019 Beaulieu-Jones B et al. *JAMA Network Open*.

Other (prototypical clustering networks, generalized low-rank models, Bayesian simulation, deep Q learning, kernel methods, mutual information-based methods, multiple instance learning, statistical methods, Gibbs sampling)	19 (11.45%)

Each paper can only be in one category. Percentage of the total number of papers shown in parentheses.

© 2019 Beaulieu-Jones B et al. *JAMA Network Open*.

eFigure. Number of Topics (k) vs Perplexity as Measured on a Hold-out Set of 10% (n=17) of the Papers Showing Perplexity Minimized at k=12

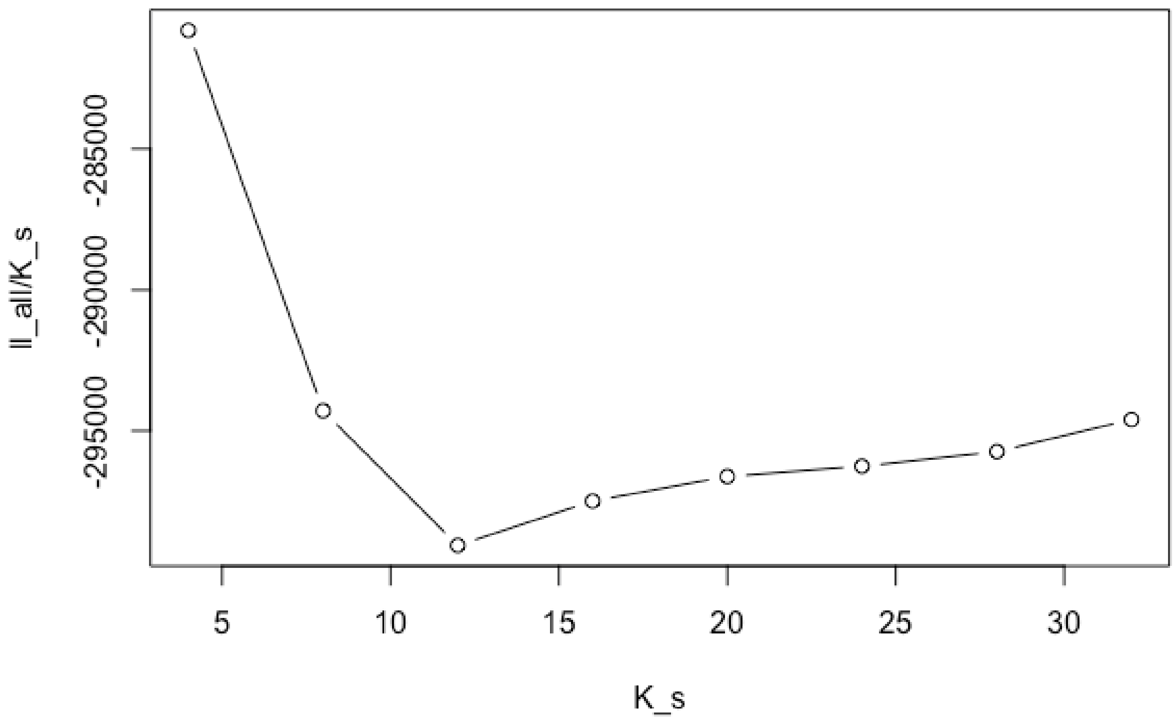

© 2019 Beaulieu-Jones B et al. *JAMA Network Open*.

eReferences

1. Jack CR Jr, Bernstein MA, Fox NC, et al. The Alzheimer's Disease Neuroimaging Initiative (ADNI): MRI methods. *J Magn Reson Imaging*. 2008;27(4):685-691.

2. DermNet NZ – All about the skin | DermNet NZ. https://www.dermnetnz.org/. Accessed September 2, 2019.

3. Ljosa V, Sokolnicki KL, Carpenter AE. Annotated high-throughput microscopy image sets for validation. *Nat Methods*. 2012;9(7):637.

4. Wang X, Peng Y, Lu L, Lu Z, Bagheri M, Summers RM. Chestx-ray8: Hospital-scale chest x-ray database and benchmarks on weakly-supervised classification and localization of common thorax diseases. In: *Proceedings of the IEEE Conference on Computer Vision and Pattern Recognition*. ; 2017:2097-2106.

5. Codella NCF, Gutman D, Emre Celebi M, et al. Skin Lesion Analysis Toward Melanoma Detection: A Challenge at the 2017 International Symposium on Biomedical Imaging (ISBI), Hosted by the International Skin Imaging Collaboration (ISIC). *arXiv [csCV]*. October 2017. http://arxiv.org/abs/1710.05006.

6. Hersh W, Müller H, Kalpathy-Cramer J. The ImageCLEFmed medical image retrieval task test collection. *J Digit Imaging*. 2009;22(6):648-655.

7. Jaeger S, Candemir S, Antani S, Wáng Y-XJ, Lu P-X, Thoma G. Two public chest X-ray datasets for computer-aided screening of pulmonary diseases. *Quant Imaging Med Surg*. 2014;4(6):475-477.

8. Shiraishi J, Katsuragawa S, Ikezoe J, et al. Development of a digital image database for chest radiographs with and without a lung nodule: receiver operating characteristic analysis of radiologists' detection of pulmonary nodules. *American Journal of Roentgenology*. 2000;174(1):71-74.

9. Bakas S, Akbari H, Sotiras A, et al. Advancing The Cancer Genome Atlas glioma MRI collections with expert segmentation labels and radiomic features. *Sci Data*. 2017;4:170117.

10. Bilic P, Christ PF, Vorontsov E, et al. The Liver Tumor Segmentation Benchmark (LiTS). *arXiv [csCV]*. January 2019. http://arxiv.org/abs/1901.04056.

11. Marcus DS, Wang TH, Parker J, Csernansky JG, Morris JC, Buckner RL. Cross-sectional MRI Data in Young, Middle Aged, Nondemented, and Demented Older Adults. http://citeseerx.ist.psu.edu/viewdoc/download?doi=10.1.1.459.2371&rep=rep1&type=pdf.

12. Johnson AEW, Pollard TJ, Berkowitz SJ, et al. MIMIC-CXR: A large publicly available database of labeled chest radiographs. *arXiv [csCV]*. January 2019. http://arxiv.org/abs/1901.07042.

13. Demner-Fushman D, Kohli MD, Rosenman MB, et al. Preparing a collection of radiology examinations for distribution and retrieval. *J Am Med Inform Assoc*. 2016;23(2):304-310.

14. Porwal P, Pachade S, Kamble R, et al. Indian Diabetic Retinopathy Image Dataset (IDRiD): A Database for Diabetic Retinopathy Screening Research. *Brown Univ Dig Addict Theory Appl*. 2018;3(3):25.

15. Johnson AEW, Pollard TJ, Shen L, Lehman LH. MIMIC-III, a freely accessible critical care

database. *Scientific*. 2016. https://www.ncbi.nlm.nih.gov/pmc/articles/PMC4878278/.

16. Pollard TJ, Johnson AEW, Raffa JD, Celi LA, Mark RG, Badawi O. The eICU Collaborative Research Database, a freely available multi-center database for critical care research. *Sci Data*. 2018;5:180178.

17. Matthews TJ, MacDorman MF, Thoma ME. Infant Mortality Statistics From the 2013 Period Linked Birth/Infant Death Data Set. *Natl Vital Stat Rep*. 2015;64(9):1-30.

18. Pocock SJ, Ariti CA, McMurray JJV, et al. Predicting survival in heart failure: a risk score based on 39 372 patients from 30 studies. *Eur Heart J*. 2013;34(19):1404-1413.

19. Buys SS, Partridge E, Black A, et al. Effect of screening on ovarian cancer mortality: the Prostate, Lung, Colorectal and Ovarian (PLCO) Cancer Screening Randomized Controlled Trial. *JAMA*. 2011;305(22):2295-2303.

20. Hayat MJ, Howlader N, Reichman ME, Edwards BK. Cancer statistics, trends, and multiple primary cancer analyses from the Surveillance, Epidemiology, and End Results (SEER) Program. *Oncologist*. 2007;12(1):20-37.

21. Group I. The International Stroke Trial (IST): a randomised trial of aspirin, subcutaneous heparin, both, or neither among 19 435 patients with acute ischaemic stroke. *Lancet*. 1997;349(9065):1569-1581.

22. Cancer Genome Atlas Research Network, Weinstein JN, Collisson EA, et al. The Cancer Genome Atlas Pan-Cancer analysis project. *Nat Genet*. 2013;45(10):1113-1120.

23. Atassi N, Berry J, Shui A, et al. The PRO-ACT database: design, initial analyses, and predictive features. *Neurology*. 2014;83(19):1719-1725.

24. Uzuner Ö, South BR, Shen S, DuVall SL. 2010 i2b2/VA challenge on concepts, assertions, and relations in clinical text. *J Am Med Inform Assoc*. 2011;18(5):552-556.

25. Sudlow C, Gallacher J, Allen N, et al. UK biobank: an open access resource for identifying the causes of a wide range of complex diseases of middle and old age. *PLoS Med*. 2015;12(3):e1001779.

26. Dawber TR, Meadors GF, Moore FE Jr. Epidemiological approaches to heart disease: the Framingham Study. *Am J Public Health Nations Health*. 1951;41(3):279-281.

27. Download MEDLINE/PubMed Data. February 2016. https://www.nlm.nih.gov/databases/download/pubmed_medline.html. Accessed September 2, 2019.

28. Limsopatham N, Collier N. *Normalising Medical Concepts in Social Media Texts by Learning Semantic Representation*.; 2016. doi:10.5281/zenodo.55013

29. Karimi S, Metke-Jimenez A, Kemp M, Wang C. Cadec: A corpus of adverse drug event annotations. *J Biomed Inform*. 2015;55:73-81.

30. FB15K-237 Knowledge Base Completion Dataset. Microsoft Download Center. https://www.microsoft.com/en-us/download/details.aspx?id=52312. Accessed September 2, 2019.

31. National Health and Nutrition Examination Survey Overview. https://www.cdc.gov/nchs/data/nhanes/nhanes_13_14/NHANES_Overview_Brochure.pdf.

© 2019 Beaulieu-Jones B et al. *JAMA Network Open*.

32. Juurlink D, Preyra C, Croxford R, et al. Canadian institute for health information discharge abstract database: a validation study. *ICES investigative report Institute for Clinical Evaluative Sciences, Toronto*. 2006.

33. Arango-Argoty G, Garner E, Pruden A, Heath LS, Vikesland P, Zhang L. DeepARG: a deep learning approach for predicting antibiotic resistance genes from metagenomic data. *Microbiome*. 2018;6(1):23.

34. Curtis C, Shah SP, Chin S-F, et al. The genomic and transcriptomic architecture of 2,000 breast tumours reveals novel subgroups. *Nature*. 2012;486(7403):346-352.

35. PsychENCODE Consortium, Akbarian S, Liu C, et al. The PsychENCODE project. *Nat Neurosci*. 2015;18(12):1707-1712.

36. Campbell PJ, Getz G, Stuart JM, Korbel JO, Stein LD. Pan-cancer analysis of whole genomes. *bioRxiv*. July 2017:162784. doi:10.1101/162784

37. Moody GB, Mark RG. The impact of the MIT-BIH arrhythmia database. *IEEE Eng Med Biol Mag*. 2001;20(3):45-50.

38. Willetts M, Hollowell S, Aslett L, Holmes C, Doherty A. Statistical machine learning of sleep and physical activity phenotypes from sensor data in 96,220 UK Biobank participants. *Sci Rep*. 2018;8(1):7961.

39. Van Essen DC, Smith SM, Barch DM, et al. The WU-Minn Human Connectome Project: an overview. *Neuroimage*. 2013;80:62-79.

40. Van Essen DC, Ugurbil K, Auerbach E, et al. The Human Connectome Project: a data acquisition perspective. *Neuroimage*. 2012;62(4):2222-2231.

41. Fan Q, Witzel T, Nummenmaa A, et al. MGH-USC Human Connectome Project datasets with ultra-high b-value diffusion MRI. *Neuroimage*. 2016;124(Pt B):1108-1114.

42. Shah V, von Weltin E, Lopez S, et al. The Temple University Hospital Seizure Detection Corpus. *Front Neuroinform*. 2018;12:83.

43. UCI Machine Learning Repository: EEG Database Data Set. https://archive.ics.uci.edu/ml/datasets/eeg+database. Accessed September 2, 2019.

44. Shoeb AH. Application of machine learning to epileptic seizure onset detection and treatment. 2009. https://dspace.mit.edu/handle/1721.1/54669?show=full. Accessed September 2, 2019.

45. Kemp B, Zwinderman AH, Tuk B, Kamphuisen HA, Oberyé JJ. Analysis of a sleep-dependent neuronal feedback loop: the slow-wave microcontinuity of the EEG. *IEEE Trans Biomed Eng*. 2000;47(9):1185-1194.

46. Silva de Lima AL, Hahn T, de Vries NM, et al. Large-Scale Wearable Sensor Deployment in Parkinson's Patients: The Parkinson@Home Study Protocol. *JMIR Res Protoc*. 2016;5(3):e172.

47. Gilson MK, Liu T, Baitaluk M, Nicola G, Hwang L, Chong J. BindingDB in 2015: A public database for medicinal chemistry, computational chemistry and systems pharmacology. *Nucleic Acids Res*. 2016;44(D1):D1045-D1053.

© 2019 Beaulieu-Jones B et al. *JAMA Network Open*.

48. Launching the Speech Commands Dataset. Google AI Blog. https://ai.googleblog.com/2017/08/launching-speech-commands-dataset.html. Accessed September 2, 2019.

© 2019 Beaulieu-Jones B et al. *JAMA Network Open*.
